# A New Method to Correct for Habitat Filtering in Microbial Correlation Networks

**DOI:** 10.3389/fmicb.2019.00585

**Published:** 2019-03-20

**Authors:** Vanessa Brisson, Jennifer Schmidt, Trent R. Northen, John P. Vogel, Amélie Gaudin

**Affiliations:** ^1^Lawrence Berkeley National Laboratory, Berkeley, CA, United States; ^2^The DOE Joint Genome Institute, Walnut Creek, CA, United States; ^3^Department of Plant Sciences, University of California, Davis, Davis, CA, United States; ^4^Department of Plant and Microbial Biology, University of California, Berkeley, Berkeley, CA, United States

**Keywords:** microbial community, correlation network, habitat filtering, network analysis algorithm, rhizosphere

## Abstract

Amplicon sequencing of 16S, ITS, and 18S regions of microbial genomes is a commonly used first step toward understanding microbial communities of interest for human health, agriculture, and the environment. Correlation network analysis is an emerging tool for investigating the interactions within these microbial communities. However, when data from different habitats (e.g., sampling sites, host genotype, etc.) are combined into one analysis, habitat filtering (co-occurrence of microbes due to habitat sampled rather than biological interactions) can induce apparent correlations, resulting in a network dominated by habitat effects and masking correlations of biological interest. We developed an algorithm to correct for habitat filtering effects in microbial correlation network analysis in order to reveal the true underlying microbial correlations. This algorithm was tested on simulated data that was constructed to exhibit habitat filtering. Our algorithm significantly improved correlation detection accuracy for these data compared to Spearman and Pearson correlations. We then used our algorithm to analyze a two real data sets of 16S variable region amplicon sequences that were expected to exhibit habitat filtering. Our algorithm was found to effectively reduce habitat effects, enabling the construction of consensus correlation networks from data sets combining multiple related sample habitats.

## Introduction

The importance of microbial communities has become increasingly recognized in a variety of contexts, including human health, agricultural sustainability, and the conservation of our natural environment and resources. While many studies have evaluated microbial community composition, generally through amplicon (16S, ITS, 18s) based sequencing approaches, an increasing number of studies address community structure and interactions through microbial correlation network analyses. Microbial correlation networks have been used to investigate microbial communities in a wide range of systems including oceans, human microbiomes, and soil and plant associated microbial communities (Barberán et al., 2012; Faust et al., 2012; Lima-Mendez et al., 2015; Milici et al., 2016; Shi et al., 2016; Delgado-Baquerizo et al., 2018).

Microbial correlation network analysis goes beyond simple surveys of community composition, and can provide important insights into the ecological interactions within microbial communities. These analyses can be used to investigate the interactive structure of microbial communities across different experimental conditions and how communities change over time. For instance, a study of bulk soil and rhizosphere microbiomes from wild oat (*Avena fatua*) found greater complexity in the rhizosphere correlation networks as compared to bulk soil, with increasing network complexity over the course of plant growth that was repeatable over two growth cycles (Shi et al., 2016). Increasing network complexity has been interpreted to indicate increased ecological interactions such as mutualistic metabolic cross-feeding and niche sharing (Berry and Widder, 2014; Shi et al., 2016). Microbial correlation networks have also been used to identify keystone species that appear to play a central role in microbial community structure. For instance, Shi et al. (2016) evaluated within and between module connectivity of network nodes to try to identify hub species, while Berry and Widder found that high degree centrality, high closeness centrality, and low betweenness centrality were indicative of keystone species in microbial correlation networks (Berry and Widder, 2014). Correlation analyses have also been used to generate testable hypotheses about specific interactions. In a study of plankton communities, an interaction between acoel flatworms (*Symsagittifera* sp.) and a green microalgae (*Tetraselmis* sp.) was predicted based on correlation analysis, and subsequently confirmed via microscopy (Lima-Mendez et al., 2015).

Several different approaches have been used to construct microbial correlation networks. While some studies use Spearman or Pearson correlations between microbial abundances, other tools have been introduced to address the particular issues associated with microbial community analysis (Deng et al., 2012; Friedman and Alm, 2012; Kurtz et al., 2015; Weiss et al., 2016; Faust and Raes, 2016). For instance, because the data represent relative abundances, these data are inherently compositional: an increase in the relative abundance of one taxon is necessarily accompanied by a decrease in the relative abundance of other taxa. Compositionality can also induce spurious correlations, a problem that is especially important in lower diversity systems (Friedman and Alm, 2012). Tools such as SparCC and SPEIK-EASI have been introduced specifically to address compositional effects in these analyses (Aitchison, 1982; Friedman and Alm, 2012; Kurtz et al., 2015; Knight et al., 2018). A comparison of network construction approaches details pros and cons of several of these approaches for detecting particular types of interactions (Weiss et al., 2016).

**FIGURE 1:**
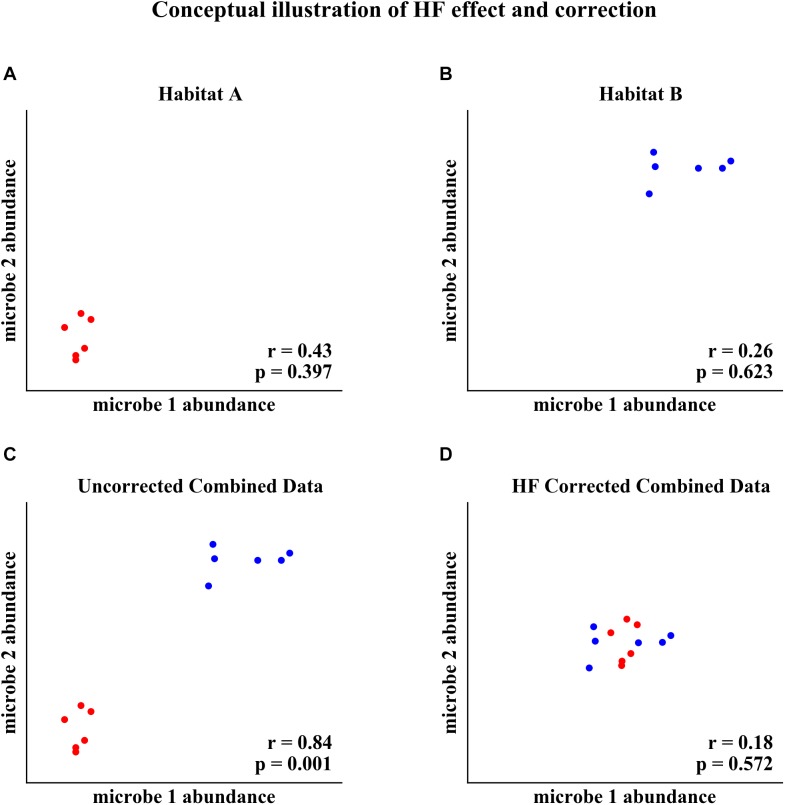
Conceptual illustration of HF effect and correction. **(A)** Two microorganisms’ abundances in Habitat A. **(B)** The same microorganisms’ abundances in Habitat B. **(C)** Combined data from Habitat A and Habitat B, resulting in a habitat induced correlation. **(D)** Correction for HF by subtracting the within habitat mean abundance eliminates detection of the spurious correlation.

Habitat filtering (HF) can confound microbial correlation network analysis when samples from different habitats (e.g., sampling sites, soil compartments, host genotype) are combined (Berry and Widder, 2014; Röttjers and Faust, 2018). HF occurs when microorganisms’ abundances are correlated with different habitats. For example, two microorganisms that are correlated with habitat will appear to be correlated with each other when data from different habitats are combined into one analysis, as conceptualized in Figure 1. The two microorganisms’ abundances are not correlated in Habitat A or in Habitat B, but both increase in abundance with the change in habitat between A and B (Figure 1A,B). When data from Habitat A and Habitat B are combined, the two microorganisms appear to be strongly correlated (Figure 1C). However, this correlation is the result of HF rather than an underlying interaction between the organisms. Correction for HF, as proposed here, eliminates detection of the spurious correlation (Figure 1D).

Habitat effects have been shown to dominate network structures when data from different habitats are combined into a single analysis. For instance, an analysis of data from the human microbiome project found that the network clustered largely by sample site on the human body (Faust et al., 2012). Similarly, a recent study of soil bacteria across a range of habitats found that bacterial habitat preference dominated co-occurrence network structure (Delgado-Baquerizo et al., 2018).

Some studies have tried to identify habitat related effects in correlation network analyses, generally by addressing the impacts of specific measurable habitat factors. In a study of plankton communities, several factors including temperature, mixed layer depth, and phosphate and nitrite concentrations were shown to have significant effects on microbial abundances and induce network correlations (Lima-Mendez et al., 2015). In that study, the authors analyzed “taxon-taxon-environment associations” in which two taxa correlated with each other and with a particular environmental factor, and used that analysis to remove some spurious correlations from the network (Lima-Mendez et al., 2015). Another study proposes an algorithm to determine global interaction coefficients that account for gradients of environmental factors (Shang et al., 2017). However, both of these analyses were facilitated by relatively large sample sizes (313 samples and 150 samples respectively) and also did not account for additional environmental factors that were not measured experimentally but may be of importance for community composition and structure (Lima-Mendez et al., 2015; Shang et al., 2017). For example, plant root exudates are complex mixes of organic compounds that have been shown to influence microbial community composition in the rhizosphere (Zhalnina et al., 2018). Even closely related plant genotypes can differ in exudate composition, which may influence microbial community composition (Iannucci et al., 2017). Thus, failing to account for HF, due to plant genotype for instance, may bias networks with spurious correlations.

In this study we propose and test a new algorithm for HF correction that could be used for microbial network analysis across multiple study systems. The aims of this approach are (a) to enable the construction of accurate consensus networks from multiple habitats by removing habitat induced edges, (b) to do this with relatively small sample sizes, and (c) when differences between habitats are not necessarily easily characterized.

## Results

### An Algorithm for HF Correction

We developed an algorithm that corrects for HF effects in microbial abundance data prior to correlation detection, enabling more accurate and unbiased identification of the underlying microbial correlations (Figure 1D). In these data sets, microorganism abundances are approximated by the (relative) abundances of amplicon sequence variants (ASVs; alternately operational taxonomic units or OTUs) in the sequencing data. The HF correction algorithm subtracts the within habitat mean abundance for each ASV from the abundances for that ASV in each sample. Thus, for a data matrix of abundance data A, the elements of the corresponding HF corrected matrix C are given by Equation 1.

(1) $${\text{C}}_{\text{ij}}={\text{A}}_{\text{ij}}-\frac{1}{\text{n}}\sum_{{\text{k in H}}_{\text{i}}}{\text{A}}_{\text{kj}}$$

Here A_ij_ represents the abundance of ASV j in sample i, H_i_ represents the set of all samples for the habitat from which sample i originated, and n represents the number of samples in H_i_. Once correction has been performed, microbial correlations can be detected with either Spearman or Pearson correlations based on the corrected data matrix C. For the purposes of the analyses below, we have used Spearman correlations after HF correction.

In order to reduce correlations caused by the compositionality of relative abundance data, the data can be transformed to reduce compositionality effects prior to HF correction and correlation detection. For the analysis of real data described here, the centered log ratio transformation was used to account for compositionality (Aitchison, 1982).

### HF Correction Improves Accuracy of Correlation Detection for Simulated Data

We tested the performance of Pearson correlations, Spearman correlations, and the HF correction algorithm in detecting correlations in simulated data. Simulated data sets represented relative abundances of 50 ASVs, with each pair of ASVs having a 10% chance of having a true correlation, and each ASV having a 10% chance of being impacted by HF. Fifty simulations were evaluated at each of a range of sample sizes (6 to 60 samples = 3 to 30 samples per habitat) and HF strengths (0 to 10 times the ASV abundance standard deviation).

Two measures were used to evaluate algorithm performance on simulated data. First, we evaluated the root mean squared error (RMSE) of the detected correlation matrix as compared to the correlation matrix used in simulated data generation prior to the imposition of the HF effect. This measure of correlation accuracy has been used previously to evaluate the performance of correlation detection algorithms to recover correlation patterns from simulated data (Friedman and Alm, 2012).

Second, the proportion of correlations detected that corresponded to true correlations in the simulated network was used as another evaluation of correlation accuracy. This was determined based on including all correlations detected with a significance cutoff of *p* < 0.01. These were compared to the randomly generated set of correlations from the initial stage of data simulation. Detected correlations that corresponded to true correlations with the same direction were considered correct, while those corresponding to no correlation or with the incorrect correlation direction were considered incorrect.

**FIGURE 2:**
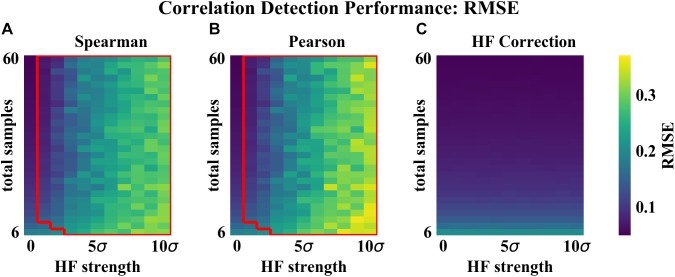
Correlation detection performance for simulated data: correlation matrix RMSE. Heat map colors represent the mean correlation matrix RMSE based on 50 simulated data sets for each combination of HF strength and sample size. **(A)** Spearman correlations. **(B)** Pearson correlations. **(C)** HF correction. Red box in **(A,B)** indicates the set of conditions (HF strength and sample size) for which there was a statistically significant improvement for HF correction compared to the other method tested.

**FIGURE 3:**
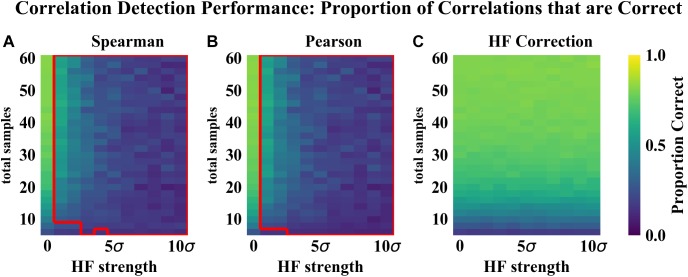
Correlation detection performance for simulated data: proportion of detected correlations that are correct. Heat map colors represent the mean proportion of correct correlations based on 50 simulated data sets for each combination of HF strength and sample size. **(A)** Spearman correlations. **(B)** Pearson correlations. **(C)** HF correction. Red box in **(A,B)** indicates the set of conditions (HF strength and sample size) for which there was a statistically significant improvement for HF correction compared to the other method tested.

**FIGURE 4:**
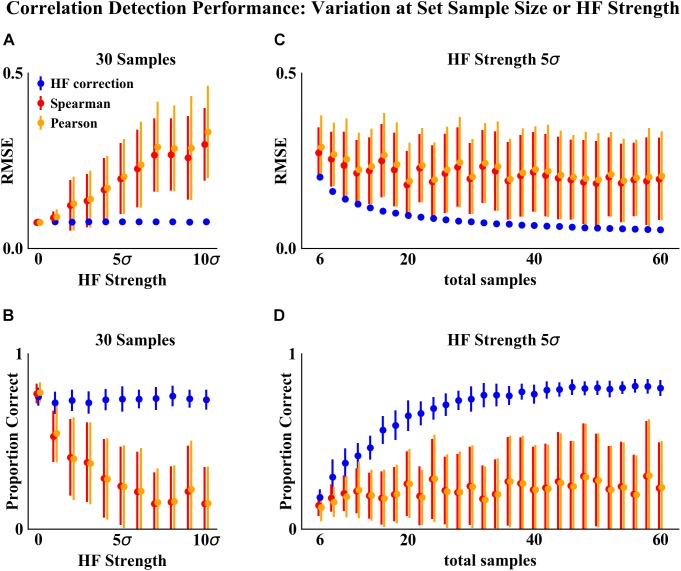
Correlation detection performance and variation on simulated data at set sample size or HF strength. **(A,B)** Set simulated sample size of 30 samples (15 samples for each habitat) and varying HF strength. **(C,D)** Set HF strength of 5σ and varying simulated sample size. Performance was measured based on two criteria: **(A,C)** RMSE between detected and simulated correlation matrices. **(B,D)** Proportion of correlations detected (at a significance cutoff of *p* < 0.01) corresponding to the initial randomly simulated correlations. Points indicate the mean and error bars indicate the standard deviation of 50 simulations.

As compared to the other correlation detection methods tested (Spearman and Pearson), HF correction resulted in significantly lower RMSE and higher proportions of correlations correct for simulated data with HF (Figure 2–4). When no HF was imposed on the simulation (corresponding to 0 on the x axes of Figure 2, 3, 4A,B), all methods demonstrated similar performance. However, even a low level of HF (HF effect size equal to the standard deviation of the data) resulted in worse performance for Spearman and Pearson correlation networks, while the performance of HF correction remained stable. The difference in performance increased with increasing strength of the HF effect. When tested over a range of sample sizes and HF strengths, these effects were statistically significant (*p* < 0.05) for all combinations except when both sample size and HF strength were very low, as indicated by the red boxes in Figure 2, 3. With either larger sample size or larger HF effect size, performance with HF correction was significantly better than that of the other methods.

### HF Correction Reduces Habitat Preference Bias in Microbial Correlation Networks

In order to assess the HF correction algorithm’s performance on real microbiome data, we analyzed two different data sets expected to exhibit significant HF effects. The first of these data sets consisted of 16S-V4 amplicon sequences from rhizosphere soil collected from two distinct maize accessions grown in three differently managed soils. The two maize accessions were Mo17 (an inbred line that was sequenced to produce a maize reference genome) (Yang et al., 2017), and DeKalb2015 (a modern commercially successful transgenic hybrid line released in 2015). All three soils were from the Century Experiment at the Russell Ranch Sustainable Agriculture Facility (University of California, Davis), from replicated plots under different management practices and crop rotations for over 20 years (Wolf et al., 2018). The three soils’ histories are described in Table 1.

**Table 1:** Soils used in experiment to explore rhizosphere microbial communities.

	Fertilization	Irrigation	Crop rotation
Soil 1: Conventional	Synthetic fertilizer	Drip irrigation	Tomato/corn
Soil 2: Organic	Compost and cover crop	Drip irrigation	Tomato/corn
Soil 3: No input, marginal	Unfertilized	Rain-fed	Wheat/fallow

The second data set was publicly available data from the National Institutes of Health Human Microbiome Project (HMP) (Consortium et al., 2012a,b). We analyzed a subset of the data that included the samples from a single body site (throat) processed at two different sequencing centers: Baylor College of Medicine (BCM) and the J. Craig Venter Institute (JCVI).

**FIGURE 5:**
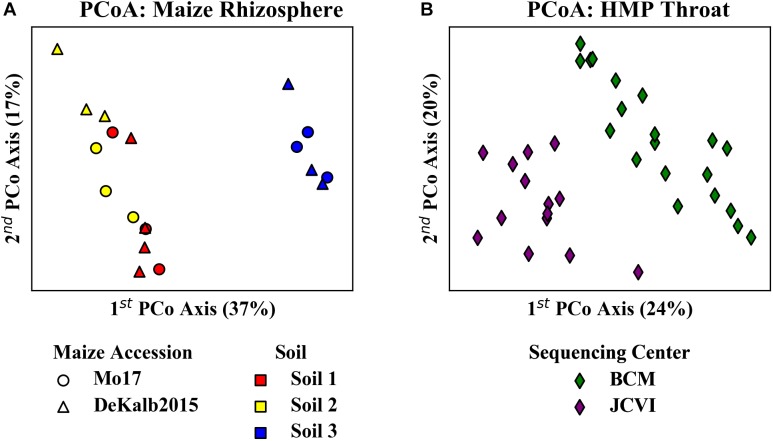
Principal coordinate analysis of **(A)** rhizosphere microbiome data from two maize accessions grown in three soils and **(B)** HMP throat microbiome data from two sequencing centers. Analysis is based on 16S-V4 sequences using Bray-Curtis distances. The clear differences in community composition apparent between the different **(A)** soils and **(B)** sequencing centers imply that HF occurs between soils.

Principal coordinate analyses of the relative abundances of ASVs/OTUs from these experiments suggested potentially strong HF effects between sample subsets from different habitats. Analysis of the maize data set revealed clear distinctions between rhizosphere communities originating from two different maize accessions grown in the three different soils (Figure 5A). There was a particularly clear difference between the communities from Soil 3 as compared to the other soils along the first principal coordinate axis. Analysis of the HPM data revealed distinctions between samples from BCM and JCVI (Figure 5B).

Individual networks were constructed for each of the two maize accessions and for the HMP throat samples using Spearman correlations, Pearson correlations, and the proposed HF correction algorithm. Data were centered log ratio transformed prior to HF correction and/or correlation detection in order to account for inherent compositionality effects in microbiome data. Correlations were included in the network based on a maximum *p*-value of 0.01. For the maize samples, and additional criterion of a minimum correlation strength of 0.75.

An independent differential abundance analysis was conducted on the same data sets to identify the habitat preference of individual ASVs/OTUs in the microbiome communities (see Methods section for details of this analysis). Any ASVs/OTUs that had a significantly (*p*_adjusted_ ≤ 0.05) increased abundance in samples from one (or two) of the maize soils or HMP sequencing centers as compared to the other(s) were considered to prefer the habitat(s). This information was used to evaluate how strongly network structures were influenced by HF effects.

**FIGURE 6:**
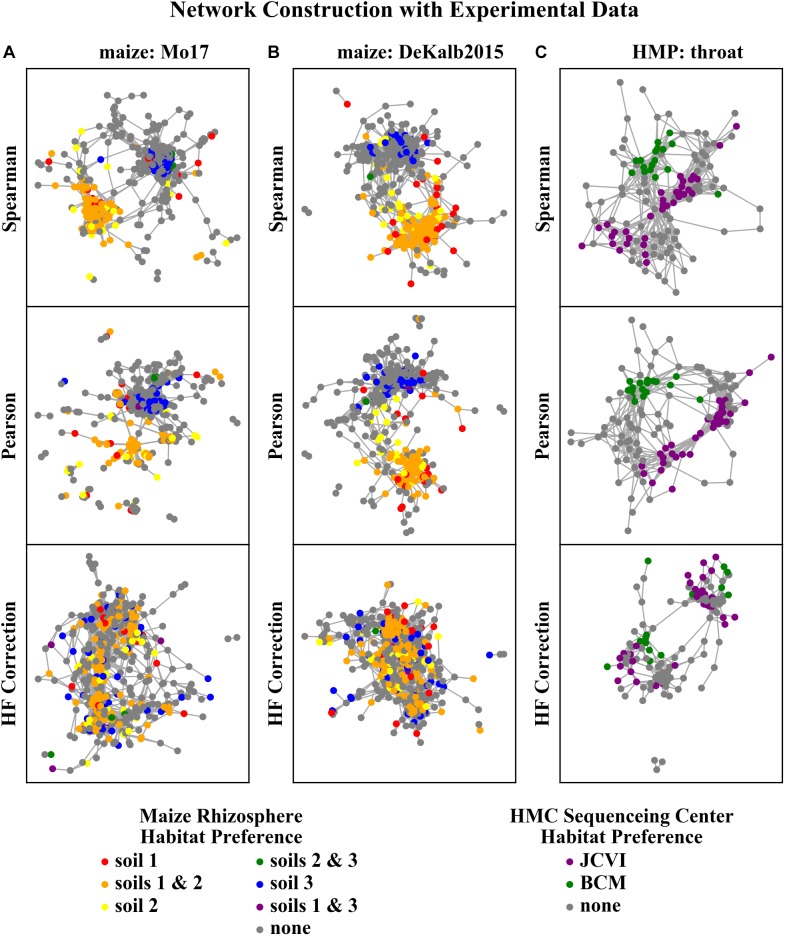
Rhizosphere microbiome correlation networks constructed with different correlation detection algorithms for **(A)** maize Mo17, **(B)** maize DeKalb2015, and **(C)** HMP throat microbiome data sets. Each node in the network represents a single ASV/OTU. ASVs/OTUs are colored based on their habitat preference as assessed by the independent differential abundance analysis.

The networks constructed with Spearman or Pearson correlations were dominated by HF effects, while those constructed with HF correction exhibited a clear reduction or elimination of these effects (Figure 6). ASVs with the same habitat preference as determined by the independent differential abundance analysis (indicated by node color in Figure 6) clustered together in the Spearman and Pearson correlation networks. This indicates that HF effects rather than underlying microbial interactions dominate the structure of these networks. However, the networks constructed with the HF correction algorithm do not exhibit these patterns.

**FIGURE 7:**
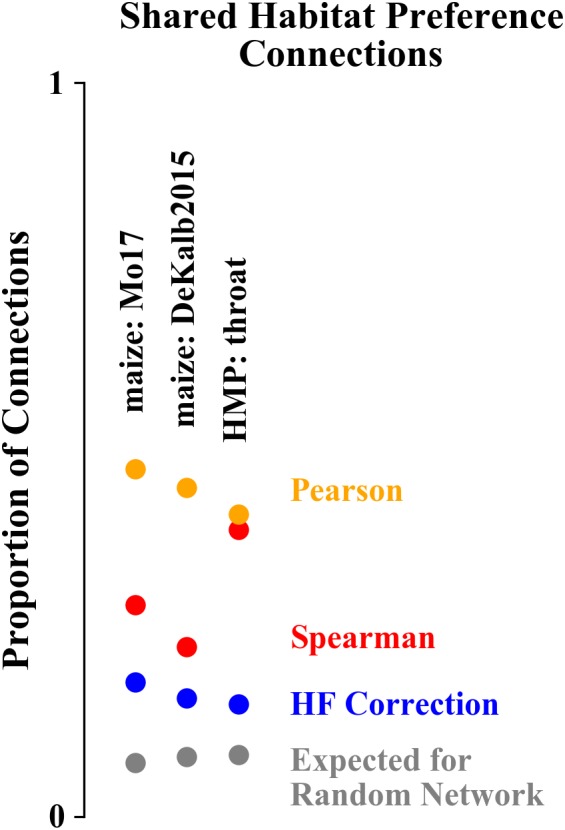
Proportion of network correlations that are between ASVs/OTUs with a shared habitat preference. Networks constructed with Pearson correlations have the highest proportion of shared habitat preference correlations while HF corrected networks have the lowest.

We quantified the extent to which the network structure corresponds to habitat preference using the proportion of network connections that are between ASVs/OTUs with a shared habitat preference (Figure 7). For networks constructed for both data sets, the Pearson correlation networks had the highest proportion of correlations between ASVs with shared habitat preference (47, 45, and 41% for Mo17, DeKalb2015, and HMP). Spearman correlation networks were intermediate for the maize rhizosphere data set (29 and 23% for Mo17 and DeKalb2015), but comparable to the Pearson correlation network for the HMP data set (39%). HF corrected networks had the lowest levels of shared habitat preference correlations (18, 16, and 15%). The differences in habitat preference correlations between network construction algorithms were statistically significant (*p* < 0.01 based on chi-square test for equality of proportions), with the exception of Spearman and Pearson correlation networks for the HMP data set, which did not differ significantly. As a point of reference, for a randomized network based on either of these data sets, this proportion would be 7–8%.

## Discussion

Correlation network analysis is an important tool for understanding interactions within microbial communities, but HF can have significant impacts that should be considered when constructing, analyzing, and interpreting these networks. HF effects are known to influence correlation network structure, and have been shown to affect a range of commonly used network construction approaches (Berry and Widder, 2014; Röttjers and Faust, 2018). In the analysis of rhizosphere microbiome sequencing data presented here, the uncorrected networks showed a strong impact of HF on the network structure (Figure 6, 7). This is consistent with the expectations based on both the soil history (Table 1) and the PCoA of the microbial community composition (Figure 5). Both crop rotation and fertilization practices are expected to influence the bulk soil microbial community, which in turn impacts the community that plants recruit to the rhizosphere from that soil. Another important factor affecting rhizosphere microbiomes is nutrient and water availability, which are strongly affected by soil management practices, and is also expected to influence rhizosphere microbiome recruitment by the plant (Koyama et al., 2014; Naylor et al., 2017).

We developed and tested an algorithm to correct for HF effects in correlation network construction when samples from different habitats are combined. The algorithm accomplishes the correction by subtracting the within habitat mean abundance from each ASV within each sample (Equation 1). This is conceptually similar to hierarchical regression, which has been used to analyze wood decay fungi co-occurrence (presence/absence) data to account for host tree preference when analyzing positive or negative associations between fungi (Ovaskainen et al., 2010).

The analysis of performance on simulated data showed that when HF was present, the HF correction was able to improve correlation detection compared to Spearman or Pearson correlations even at small sample sizes (*n* = 6 total samples (3 per habitat) for most HF strengths tested) (Figure 2, 3). Performance, measured as low RMSE and a high proportion of correct correlations, increased with increasing sample size up to approximately 30 total samples (15 per habitat), at which point performance leveled off (Figure 1–3). This is consistent with another study which recommended a sample size of at least 25 samples based on reduced specificity of network correlations at lower sample sizes (Berry and Widder, 2014).

Increasing HF strength negatively impacted the performance of Spearman and Pearson correlations for network construction. However, HF correction was able to maintain consistent performance, and lower variability in performance, across the full range of HF strengths tested. A recent comparison of several microbial correlation network construction methods, including CoNet (Faust and Raes, 2016), SparCC (Friedman and Alm, 2012), SPEIC-EASI (Kurtz et al., 2015), and Spearman correlations, also found that all methods tested exhibited reduced precision on data with simulated HF (Röttjers and Faust, 2018).

Selection of appropriate methods for correlation network analysis requires careful consideration of the advantages and limitations of different approaches with respect to the dataset being analyzed. For instance, the method presented in this study assumes monotonic correlations, and other correlation patterns would not be correctly detected. The Maximal Information Coefficient method has been studied to detect non-monotonic correlations in microbial community analysis, but that method does not address HF effects (Reshef et al., 2011; Weiss et al., 2016). Compositionality of the data is another potential concern. Moderate compositional effects can be reduced by the CLR transform and Spearman correlations (Friedman and Alm, 2012; Weiss et al., 2016), which are incorporated into the HF correction analysis presented here. However, low diversity, highly compositional data may require an approach specifically designed to address that problem, such as the SparCC program (Friedman and Alm, 2012). These types of tradeoffs should be considered when selecting an appropriate analysis method.

HF correction is most appropriate when HF is known or suspected to have a significant impact based on preliminary analyses, such as the habitat related differences revealed in the PCoA plots in Figure 5. The algorithm presented here is limited to correction for habitats defined in the analysis, and care must be taken to identify the appropriate habitat groups ahead of analysis. Additionally, different habitat groups should have similar sample sizes. If sample sizes differ significantly, results could be skewed to favor interactions associated with a particular habitat.

Besides direct mutualistic (or antagonistic) interactions between microorganisms, other mechanisms can contribute to positive (or negative) correlations once HF effects have been corrected. For instance, if two microorganisms A and B both individually have positive interactions with a third microorganism C, then A and B will appear to have a positive correlation with each other, even if they do not interact directly, a situation referred to as conditional independence. Most correlation network construction algorithms cannot differentiate this from correlations due to direct interactions, although the SPIEC-EASI method was developed to try to address this concern in microbial networks (Kurtz et al., 2015). Similarly, it is also possible that a correlation between microorganisms will be due to environmental factors not accounted for in the HF correction, such as the availability of a particular substrate used by both microorganisms.

Understanding and accounting for HF effects is an important part of conducting meaningful microbial correlation network analyses of data from varying habitats. HF correction accounts for those effects and allows for the construction of consensus networks representing the underlying microbial correlations from data spanning multiple related habitats.

## Materials and Methods

### Simulated Data Generation

Data simulation was based on that described by Friedman et al., with some modifications (Friedman and Alm, 2012). Simulated data were generated for 50 ASVs, at a range of HF strengths (0–10σ) and a range of sample sizes (6–60 samples), with half of the samples (3–30) belonging to each of 2 simulated habitats. To generate an initial set of correlations, any pair of ASVs was given a 10% chance of being perfectly correlated, with equal probabilities that correlations were positive or negative. Based on those correlations, the nearest valid covariance matrix was determined based on a standard deviation of 0.1 using the cov_nearest function in the StatsModels Python module. Additionally, each ASV was given a 10% chance of exhibiting HF, with equal probabilities of positive or negative correlation with habitat. Data were drawn from a lognormal distribution with a standard deviation of 0.1. Fifty simulated data sets were generated for each combination of HF strength and sample size.

### Maize Rhizosphere Experimental Data Collection and Analysis

Experimental data from rhizosphere soil samples were from two different maize accessions grown in three different soil types as described above. Maize seeds were surface sterilized in 5% sodium hypochlorite for one minute, germinated in petri dishes, and planted in five gallon pots containing one of the three soils. Plants were grown in a greenhouse for 8 weeks with automated drip irrigation and no fertilization. At the end of the experiment, plants were harvested and rhizosphere soil was collected as described by Barillot et al. (2013). DNA was extracted with the MoBio PowerSoil extraction kit [MoBio Laboratories Inc., Carlsbad, CA, United States] according to the manufacturer’s instructions.

16S-V4 amplicon sequencing was conducted at the DOE Joint Genome Institute using the 515F (GTGYCAGCMGCCGCGGTAA), 805R (GGACTACNVGGGTWTCTAAT) primer set. Sequencing was performed on an Illumina MySeq in 2 × 300 run mode. Sequencing data were analyzed with the DADA2 package in R based on the big data analysis pipeline with the following parameters: truncLen = (160, 120), maxEE = (2, 5), and truncQ = 2 (Callahan et al., 2016). The resulting sequence count data were analyzed using the phyloseq package in R (McMurdie and Holmes, 2013). Chloroplast and mitochondrial sequences were removed and data were rarefied to the minimum number of reads in all samples prior to further analysis.

### Network Construction

Networks were constructed with each of three different approaches: Spearman correlations, Pearson correlations, and our proposed HF correction algorithm. For HF correction networks, rhizosphere microbial data were corrected based on equation 1 and correlation were determined from corrected data using Spearman correlations. For analysis of simulated networks, both positive and negative correlations were included. In order to select significant correlations to determine the proportion of correct correlations, a significance cutoff of *p* < 0.01 was applied. For analysis of experimental data, only ASVs detected in at least 50% of the samples being analyzed were included in the analysis. Experimental data were first transformed with the centered log transform in order to account for compositionality of the data. In addition to the significance cutoff of *p* < 0.01. For the maize rhizosphere data, a correlation strength cutoff of *r* > 0.75 was also applied. Only positive correlations were included in the networks based on experimental data.

### Differential Abundance Analysis

An independent differential abundance analysis was conducted to identify the habitat preference of individual ASVs/OTUs. This analysis was conducted using DeSeq2 to compare ASV/OTU abundances between pairs of soil types (Love et al., 2014). *P*-values were adjusted to account for multiple comparisons using the Benjamini and Hochberg correction. Adjusted *p*-values below 0.05 were considered to indicate habitat preference for the associated ASV/OTU.

## Data Availability

The DNA sequencing dataset used in this study is available through the JGI Genome Portal at https://genome.jgi.doe.gov/portal with the project ID 1138452. An implementation of the HF correction algorithm as a python module, along with the maize rhizosphere ASV abundance table analyzed in this study, is available through GitHub at https://github.com/vbrisson/HabitatCorrectedNetwork. The HPM data is available as an OTU table from https://www.hmpdacc.org/HMQCP/.

## Author Contributions

VB developed and implemented the habitat filtering correction algorithm and analyzed the results. VB and JS tested the algorithm. AG and JS designed the rhizosphere experiment. JS performed the rhizosphere experiments. TN, JV, and AG provided scientific advice. VB, JS, JV, TN, and AG contributed to the discussion of the results and writing of the manuscript.

## Conflict of Interest Statement

The authors declare that the research was conducted in the absence of any commercial or financial relationships that could be construed as a potential conflict of interest.

## References

[B1] Aitchison J. (1982). The statistical analysis of compositional data. *J. R. Stat. Soc. Ser. B* 44 139–177.

[B2] Barberán A. Bates S. T. Casamayor E. O. Fierer N. (2012). Using network analysis to explore co-occurrence patterns in soil microbial communities. *ISME J.* 6 343–351. 10.1038/ismej.2011.119 21900968 PMC3260507

[B3] Barillot C. D. C. Sarde C.-O. Bert V. Tarnaud E. Cochet N. (2013). A standardized method for the sampling of rhizosphere and rhizoplan soil bacteria associated to a herbaceous root system. *Ann. Microbiol.* 63 471–476. 10.1007/s13213-012-0491-y

[B4] Berry D. Widder S. (2014). Deciphering microbial interactions and detecting keystone species with co-occurrence networks. *Front. Microbiol.* 5:219. 10.3389/fmicb.2014.00219 24904535 PMC4033041

[B5] Callahan B. J. McMurdie P. J. Rosen M. J. Han A. W. Johnson A. J. A. Holmes S. P. (2016). DADA2: high-resolution sample inference from Illumina amplicon data. *Nat. Methods* 13 581–583. 10.1038/nmeth.3869 27214047 PMC4927377

[B6] Consortium T. H. M. P. Huttenhower C. Gevers D. Knight R. Abubucker S. Badger J. H. (2012a). Structure, function and diversity of the healthy human microbiome. *Nature* 48:207. 10.1038/nature11234 22699609 PMC3564958

[B7] Consortium T. H. M. P. Methé B. A. Nelson K. E. Pop M. Creasy H. H. Giglio M. G. (2012b). A framework for human microbiome research. *Nature* 486:215. 10.1038/nature11209 22699610 PMC3377744

[B8] Delgado-Baquerizo M. Oliverio A. M. Brewer T. E. Benavent-González A. Eldridge D. J. Bardgett R. D. (2018). A global atlas of the dominant bacteria found in soil. *Science* 359 320–325. 10.1126/science.aap9516 29348236

[B9] Deng Y. Jiang Y.-H. Yang Y. He Z. Luo F. Zhou J. (2012). Molecular ecological network analyses. *BMC Bioinformatics* 13:113. 10.1186/1471-2105-13-113 22646978 PMC3428680

[B10] Faust K. Raes J. (2016). CoNet app: inference of biological association networks using Cytoscape. *F1000Research* 5:1519. 10.12688/f1000research.9050.2 27853510 PMC5089131

[B11] Faust K. Sathirapongsasuti J. F. Izard J. Segata N. Gevers D. Raes J. (2012). Microbial co-occurrence relationships in the human microbiome. *PLoS Comput. Biol.* 8:e1002606. 10.1371/journal.pcbi.1002606 22807668 PMC3395616

[B12] Friedman J. Alm E. J. (2012). Inferring correlation networks from genomic survey data. *PLoS Comput. Biol.* 8:e1002687. 10.1371/journal.pcbi.1002687 23028285 PMC3447976

[B13] Iannucci A. Fragasso M. Beleggia R. Nigro F. Papa R. (2017). Evolution of the crop rhizosphere: impact of domestication on root exudates in tetraploid wheat (*Triticum turgidum L.*). *Front. Plant Sci.* 8:2124. 10.3389/fpls.2017.02124 29326736 PMC5733359

[B14] Knight R. Vrbanac A. Taylor B. C. Aksenov A. Callewaert C. Debelius J. (2018). Best practices for analysing microbiomes. *Nat. Rev. Microbiol.* 16 410–422. 10.1038/s41579-018-0029-9 29795328

[B15] Koyama A. Wallenstein M. D. Simpson R. T. Moore J. C. (2014). Soil bacterial community composition altered by increased nutrient availability in Arctic tundra soils. *Front. Microbiol.* 5:516. 10.3389/fmicb.2014.00516 25324836 PMC4183186

[B16] Kurtz Z. D. Müller C. L. Miraldi E. R. Littman D. R. Blaser M. J. Bonneau R. A. (2015). Sparse and compositionally robust inference of microbial ecological networks. *PLoS Comput. Biol.* 11:e1004226. 10.1371/journal.pcbi.1004226 25950956 PMC4423992

[B17] Lima-Mendez G. Faust K. Henry N. Decelle J. Colin S. Carcillo F. (2015). Ocean plankton. Determinants of community structure in the global plankton interactome. *Science* 348:1262073. 10.1126/science.1262073 25999517

[B18] Love M. I. Huber W. Anders S. (2014). Moderated estimation of fold change and dispersion for RNA-seq data with DESeq2. *Genome Biol.* 15:550. 10.1186/s13059-014-0550-8 25516281 PMC4302049

[B19] McMurdie P. J. Holmes S. (2013). phyloseq: an R package for reproducible interactive analysis and graphics of microbiome census data. *PLoS One* 8:e61217. 10.1371/journal.pone.0061217 23630581 PMC3632530

[B20] Milici M. Deng Z.-L. Tomasch J. Decelle J. Wos-Oxley M. L. Wang H. (2016). Co-occurrence analysis of microbial taxa in the atlantic ocean reveals high connectivity in the free-living bacterioplankton. *Front. Microbiol.* 7:649. 10.3389/fmicb.2016.00649 27199970 PMC4858663

[B21] Naylor D. DeGraaf S. Purdom E. Coleman-Derr D. (2017). Drought and host selection influence bacterial community dynamics in the grass root microbiome. *ISME J.* 11 2691–2704. 10.1038/ismej.2017.118 28753209 PMC5702725

[B22] Ovaskainen O. Hottola J. Siitonen J. (2010). Modeling species co-occurrence by multivariate logistic regression generates new hypotheses on fungal interactions. *Ecology* 91 2514–2521. 10.1890/10-0173.1 20957941

[B23] Reshef D. N. Reshef Y. A. Finucane H. K. Grossman S. R. McVean G. Turnbaugh P. J. (2011). Detecting novel associations in large data sets. *Science* 334 1518–1524. 10.1126/science.1205438 22174245 PMC3325791

[B24] Röttjers L. Faust K. (2018). From hairballs to hypotheses–biological insights from microbial networks. *FEMS Microbiol. Rev.* 42 761–780. 10.1093/femsre/fuy030 30085090 PMC6199531

[B25] Shang Y. Sikorski J. Bonkowski M. Fiore-Donno A.-M. Kandeler E. Marhan S. (2017). Inferring interactions in complex microbial communities from nucleotide sequence data and environmental parameters. *PLoS One* 12:e0173765. 10.1371/journal.pone.0173765 28288199 PMC5348048

[B26] Shi S. Nuccio E. E. Shi Z. J. He Z. Zhou J. Firestone M. K. (2016). The interconnected rhizosphere: high network complexity dominates rhizosphere assemblages. *Ecol. Lett.* 19 926–936. 10.1111/ele.12630 27264635

[B27] Weiss S. Van Treuren W. Lozupone C. Faust K. Friedman J. Deng Y. (2016). Correlation detection strategies in microbial data sets vary widely in sensitivity and precision. *ISME J.* 10 1669–1681. 10.1038/ismej.2015.235 26905627 PMC4918442

[B28] Wolf K. M. Torbert E. E. Bryant D. Burger M. Denison R. F. Herrera I. (2018). The century experiment: the first twenty years of UC Davis’ Mediterranean agroecological experiment. *Ecology* 99:503. 10.1002/ecy.2105 29338085

[B29] Yang N. Xu X.-W. Wang R.-R. Peng W.-L. Cai L. Song J.-M. (2017). Contributions of Zea mays subspecies mexicana haplotypes to modern maize. *Nat. Commun.* 8:1874. 10.1038/s41467-017-02063-5 29187731 PMC5707364

[B30] Zhalnina K. Louie K. B. Hao Z. Mansoori N. da Rocha U. N. Shi S. (2018). Dynamic root exudate chemistry and microbial substrate preferences drive patterns in rhizosphere microbial community assembly. *Nat. Microbiol.* 3 470–480. 10.1038/s41564-018-0129-3 29556109

